# A Novel Human Anti-FV mAb as a Potential Tool for Diagnostic and Coagulation Inhibitory Approaches

**DOI:** 10.3390/ijms26062721

**Published:** 2025-03-18

**Authors:** Margherita Passariello, Rosa Rapuano Lembo, Lorenzo Manna, Ciro Miele, Antonello Merlino, Cristina Mazzaccara, Antonio Leonardi, Claudia De Lorenzo

**Affiliations:** 1 Ceinge—Biotecnologie Avanzate s.c.a.r.l., Via Gaetano Salvatore 486, 80145 Naples, Italy; margherita.passariello@unina.it (M.P.); rosa.rapuano@unimi.it (R.R.L.); lorenzo.manna@unina.it (L.M.); 2 Department of Molecular Medicine and Medical Biotechnologies, University of Naples “Federico II”, Via S. Pansini 5, 80131 Naples, Italy; 3 European School of Molecular Medicine, University of Milan, 20122 Milan, Italy; 4 UOC Laboratory Medicine of Hematology and Hemostasis, Federico II University Hospital, 80131 Naples, Italy; ciro.miele@unina.it (C.M.);; 5 Department of Biology, University of Naples “Federico II”, Complesso Universitario Monte Sant’Angelo, Via Cinthia, 26, 80126 Napoli, Italy; antonello.merlino@unina.it

**Keywords:** thrombosis, coagulation cascade, Human Factor V, thrombin, human mAbs, anticoagulant therapy

## Abstract

Cardiovascular diseases, including thrombosis, are the leading cause of mortality worldwide. The generation of monoclonal antibodies (mAb) targeting specific coagulation factors could provide more targeted and safer anticoagulant therapies. Factor V (FV) is a critical cofactor in the prothrombinase complex, which catalyzes the conversion of prothrombin to thrombin, a key enzyme in the coagulation cascade. We isolated a novel human antibody specific to FV by using phage display technology. The selection occurred by panning a large repertoire of phages expressing human antibody fragments (scFv) in parallel on the purified recombinant protein in its native form (FV) or activated by proteolytic maturation (Factor Va (FVa)). Through ELISA screening, we identified the clone with the highest binding affinity for both targets, and it was successfully converted into IgG1. The novel human mAb, called D9, was found capable of binding to Factor V with a low nM affinity both by ELISA and BLI assays, whereas its cross-reactivity with some other coagulation factors was found null or very poor. Furthermore, when tested in blood clotting tests, it was found able to prolong activated partial thromboplastin time (aPTT). Thus, D9 could become not only a potential therapeutic agent as a specific anticoagulant but also a precious tool for diagnostic and research applications.

## 1. Introduction

Cardiovascular diseases are the leading cause of mortality worldwide, representing about one-third of the registered deaths every year. There is a wide spectrum of etiological factors, both genetic and environmental, at the base of these diseases, which can have different implications and molecular mechanisms. Remarkably, many of these alterations ultimately converge into a common downstream manifestation: thrombosis [1,2]. The latter is due to the occlusion of blood vessels, triggered by a dysregulated formation of clots. To ensure a normal blood flow, the hemostatic system relies on a fine balance between procoagulant and anticoagulant forces, enabling an effective physiological response to hemorrhages while allowing the prevention of pathological thrombus formation. A disruption of this balance toward the procoagulant side can result in thromboembolic events, whereas changes toward the anticoagulant side can lead to hemorrhagic diseases. These pathophysiological processes are prompted by a complex interplay of coagulation cascade factors in conjunction with their dynamic interactions with vascular endothelium, platelets, and other circulating cellular components [3]. In patients with hemophilia A or B, characterized by a hereditary deficit of Factor VIII, or in those with an inherited hemorrhagic disease due to decreased plasma levels of Factor V (FV), characterized by mild–severe hemorrhagic symptoms, the propagation phase cannot take place, and consequently, insufficient thrombin is generated to form a stable clot [2,4]. On the other hand, mutations of FV, such as the one leading to FV Leiden resistance degrading, are responsible for hereditary thrombophilia due to the gain of function of this factor [3]. Several other imbalances can lead to this outcome, and they have been classified into three different groups of alterations named “Virchow’s triad”: endothelial injury, vascular stasis, and hypercoagulability. Notably, even cancer can cause hypercoagulability states through the overexpression of procoagulant proteins and also the interaction of the tumor itself with the blood vessel endothelium [1,5]. Hence, the causes of thrombosis are multifactorial, and the clinical phenotype is due to the combination of genetic, epigenetic, and/or acquired predisposing factors, leading to abnormal clot formation and increasing the risk of thrombotic events [6,7].

Given that blood hypercoagulability significantly contributes to thrombogenesis, modulating the coagulation cascade is crucial in the management of patients with altered hemostasis. Coagulation is a complex series of enzymatic reactions that culminate in the formation of a blood clot. This intricate cascade involves the activation of clotting factors in a precisely regulated sequence, ensuring rapid response to vascular injury while preventing unwarranted clot formation [8].

For decades, heparins (initially unfractionated and, later on, low-molecular-weight heparins) and vitamin K antagonists (warfarin, phenprocoumon, acenocoumarol) have been employed in the treatment and prevention of thromboembolism [9].

However, despite the excellent clinical outcomes achieved with traditional anticoagulants, the use of heparin (both low- and high-molecular-weight), which acts as an indirect anticoagulant by enhancing the activity of antithrombin (AT), still carries a risk of heparin-induced thrombocytopenia, and their long-term use via parenteral administration is limited in the clinical settings. Similarly, although vitamin K antagonists (VKA) are well established in treating a wide range of thromboembolic disorders, by inhibiting the synthesis of vitamin K-dependent clotting factors through vitamin K epoxide reductase inhibition, their use is hindered by several limitations, including delayed onset and offset of action, genetic variations in metabolism, and interactions with food and other drugs, thus requiring frequent monitoring and dose adjustments [10].

In the past 15 years, some researchers have been focusing on the new direct oral anticoagulants (DOACs) [11] that received FDA approval in 2010, replacing VKA thanks to their versatility, favorable pharmacological profile, and lack of need for monitoring [12,13]. Nonetheless, despite having a better safety profile than VKA, the risk of bleeding continues to be a concern, with a case fatality rate of major bleeding of 8% [14], making the development of specific antidotes necessary [15,16]. This highlights the ongoing need for more targeted and safer anticoagulant therapies.

Aptamers were also considered candidates for this aim due to their ability to bind to their target protein with high affinity and specificity and their lack of immunogenicity. Several aptamers targeting blood coagulation factors have been evaluated in clinical trials and have shown promising preclinical results. However, none of these aptamers have been approved for clinical use due to challenges related to their short half-life and rapid renal clearance [12,17,18,19].

The generation of novel human monoclonal antibodies, endowed with higher MW (155 kDa), increased stability, and binding specificity, could allow for the targeting of specific coagulation factors with minimal or absent off-target effects and interactions with other proteins in the coagulation cascade, as well as increasing the half-life in the circulation of therapeutic agents. In this regard, pro-accelerin or Factor V could be a good target as it is involved in the common pathway of the coagulation cascade. Furthermore, a mAb specific to FV could have a dual function as a procoagulant and as an anticoagulant, depending on its activatory or inhibitory effects [20]. Factor V is a critical cofactor in the prothrombinase complex, which catalyzes the conversion of prothrombin to thrombin, a key enzyme in the coagulation cascade. The specific inhibition of Factor V by an antibody in patients with altered hemostasis could significantly attenuate thrombin generation, thereby reducing clot formation and the associated risk of thrombotic events [20].

Factor V is a single-chain plasma glycoprotein containing multiple domains (A1, A2, A3, C1, C2) and the large activation peptide (B domain) between A2 and A3. It represents the precursor or pro-cofactor of Factor Va, which is generated by proteolysis and removal of the inhibitory B domain during activation of FV mediated by thrombin. FVa is the active form, which acts as a cofactor for FXa, leading to prothrombin activation essential for efficient blood clotting [20,21].

Here, we isolated a novel fully human monoclonal antibody specific to Factor V by using an innovative strategy of selection based on phage display, starting from a large human scFv-phage library [22,23]. The goal was to obtain an antibody that would recognize both Factor V and Va by parallel selections on the two targets.

The development of an anti-Factor V monoclonal antibody could be useful not only as a potential tool for therapeutic use but also for diagnostic and research applications. Indeed, it could be used for reversing conditions of hypercoagulability but also be employed in diagnostic assays to measure the levels of Factor V in human blood samples to highlight FV deficits in patients with hemorrhagic symptoms, and, finally, it could be used to purify Factor V for the development of therapeutic products.

## 2. Results

### 2.1. Isolation and Screening of Novel Human scFvs Specific to Human Factor V

**Figure 1:**
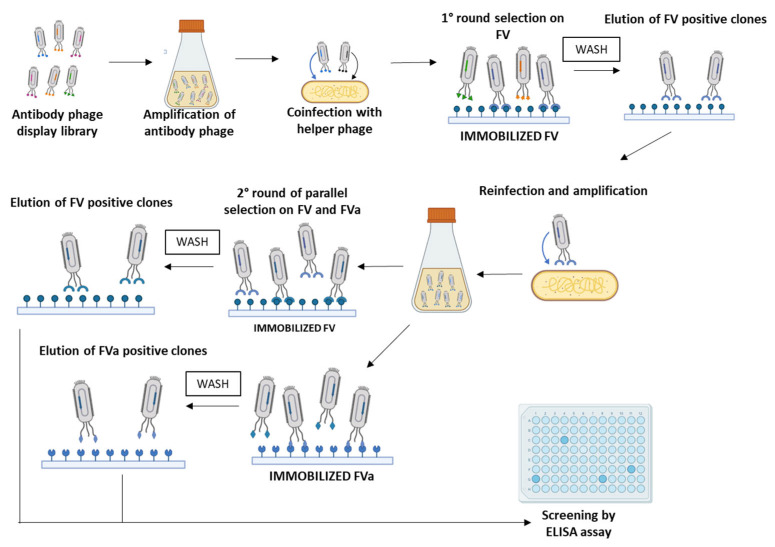
Phage display strategy for the isolation of scFvs specific to FV and FVa. The first round of selection was performed on an immobilized native FV protein, followed by washes and elution of positive clones. After amplification, the following two panning rounds were performed in parallel on immobilized FV and FVa. The identification of positive clones was carried out by screening performed by ELISA assays.

**Figure 2:**
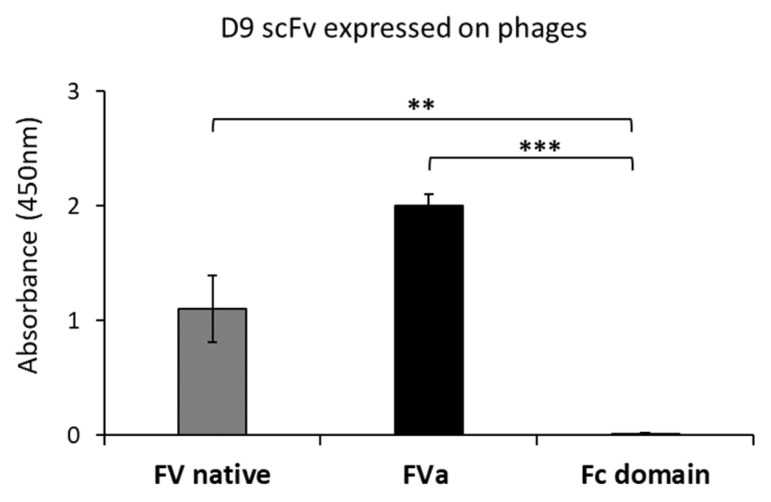
Binding by ELISA assays of D9 phage on FV and FVa. Binding assays of D9 positive phage-scFv clone by ELISA on human active FVa or FV, which were coated on the plates at a concentration of 5 µg/mL. In parallel assays, the Fc domain was used as a negative control. The binding signal was detected with an HRP-conjugated anti-M13 mAb, followed by analyses of absorbance values by using an Envision plate reader. The data were obtained as the mean of at least three determinations and presented as ±SD. The Shapiro–Wilk test was used to test the normality assumption, and *p*-values were calculated by comparing binding values of the D9 phage clone on native FV or mature FVa to that observed on the control Fc domain, respectively, and the values reported are *** *p* ≤ 0.001 and ** *p* < 0.01, which were obtained by Student’s *t*-test (two variables). Supplementary information (raw data) for Figure 2 is reported in the Supplementary Materials as Table S1.

In order to isolate an scFv specific to FVa, we designed a selection strategy based on phage display technology, starting from a large human scFv phage library [22,23]. In particular, to select a scFv able to specifically recognize both the precursor FV and the activated cofactor FVa, we planned parallel panning rounds on human purified target proteins, either in native or activated form, derived from human blood plasma. In particular, the first panning round of the phage library was performed on immobilized native human Factor V; then, the phages were eluted by the classical acidic elution method by lowering the pH and used to infect *E. Coli* TG1 cells for amplification. The amplified pool of eluted phages was subjected to two consecutive parallel panning rounds performed on human FV or human FVa (Figure 1) to increase the possibility of obtaining a common sequence by the two selections capable of recognizing both protein forms. The screening of positive binders was performed by massive parallel ELISA assays on the two immobilized proteins. We identified a positive clone, named D9, isolated through the combination of selection rounds on native and mature forms, endowed with high specificity for both the FV and the active FVa. The D9 clone displayed on phages was identified from the third round on FVa and tested in triplicates on immobilized native FV or active FVa protein by ELISA assays, as well as on an unrelated protein, such as the human Fc domain, used as a negative control, to verify the specificity of the binder for FV. As shown in Figure 2, no significant binding to the Fc was observed, whereas a strong binding signal was detected on both FV and FVa, thus confirming the efficiency of the selection strategy used.

The cDNA from the phage clone encoding the selected scFv was extracted by digestion with suitable restriction enzymes to confirm the presence of a full-size insert and then fully sequenced, as described in the Materials and Methods.

### 2.2. Expression of D9 Clone as Soluble scFv

**Figure 3:**
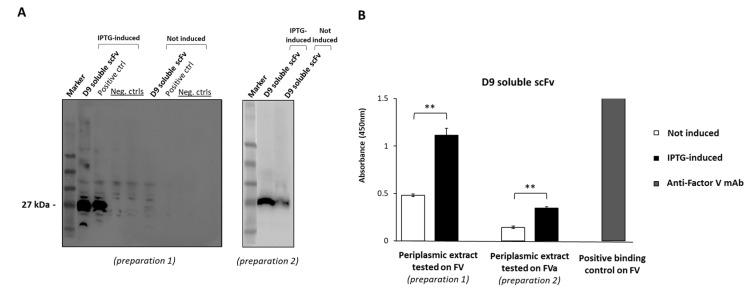
Analysis of D9 as soluble scFv and conversion into full-size mAb. (**A**) Western Blotting analyses of two different periplasmic extracts (preparation 1 and preparation 2) of bacterial cells transformed with D9 clone, expressed in the absence (not induced) or in the presence (induced) of IPTG, used for induction by incubation O.N. at 25 °C. The signal was detected by using the HRP-conjugated anti-cmyc antibody (from Miltenyi Biotec, Bergisch Gladbach, Germany). The blot images were acquired by using the ChemiDoc Imaging System and optimized by Image Lab software (version 6.0.1). The full-length blot images are reported in theadditional file. (**B**) In parallel, the periplasmic extracts (preparations 1 and 2) were tested on immobilized human FV- or FVa-purified protein by ELISA assays, and the binding was detected by using the HRP-conjugated anti-cmyc antibody. As a positive control, a commercial anti-FV mAb was used at a concentration of 200 nM to measure the max signal of immobilized FV protein. Data were obtained by two determinations and presented as mean ± SD. The Shapiro–Wilk test was used to test the normality assumption, and *p*-values were calculated by comparing the binding to FV-native or FVa-immobilized proteins of IPTG-induced D9 soluble scFv to that of the not-induced culture for each preparation. The value reported is ** *p* < 0.01, which was obtained by Student’s *t*-test (two variables). Supplementary information (raw data) for Figure 3B is reported in the Supplementary Materials as Table S2.

We investigated whether the novel identified scFv could be expressed as a soluble protein and could retain the binding properties of the scFv displayed on phages. To this aim, the cDNA encoding the scFv was used to transform the bacterial strain SF110, and the expression was induced by adding Isopropyl β-d-1-thiogalactopyranoside (IPTG) overnight (O.N.) at 25 °C. The periplasmic extracts of the selected clone, before and after the induction with IPTG, were first analyzed by Western Blotting (WB) and then tested by ELISA assays by using an anti-cmyc antibody. As shown in Figure 3A, the novel scFv was expressed as a soluble protein of the expected molecular weight of 27 kDa. Two different periplasmic extracts (preparations 1 and 2), after IPTG induction, were then tested to verify D9 binding specificity forhuman immobilized FV- or FVa-purified protein (Figure 3B), which fully confirmed its ability to bind to both the proteins even when the scFv was expressed as a soluble protein. On the other hand, no binding was observed with the periplasmic extract that was not induced with IPTG, thus confirming that the binding to FV is mediated only by the expressed exogenous sequence cloned downstream of the IPTG-inducible promoter. The higher absorbance value obtained for FV compared to FVa was likely due to the higher scFv expression level (and consequent concentration) in preparation 1 tested on FV.

### 2.3. The Binding of D9 to FV: An In Silico Approach

**Figure 4:**
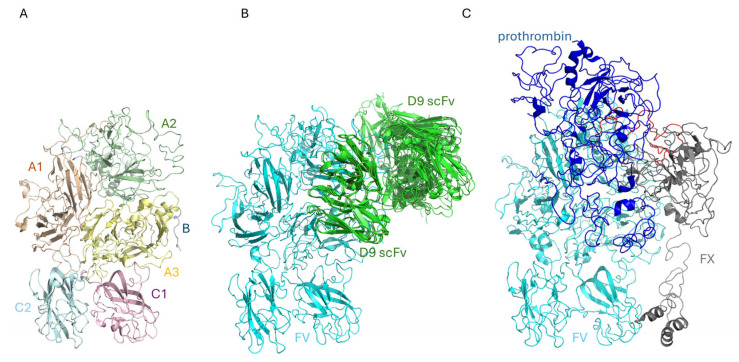
In silico binding of D9 scFv to FV. Overall structures of FV (PDB code 7KVE [26], panel **A**) of the complex between FV and D9 scFv from docking calculations (panel **B**) and of the complex of FV with FX and prothrombin (PDB code 7TPP [28], panel **C**). In panel (**A**), the A1, A2, A3, B, C1, and C2 domains are in white, pale green, light blue, pale yellow, light pink, and pale cyan, respectively. In panel (**B**), FV is in cyan. Eight out of the ten poses with the highest scores in the docking calculation are shown. In this panel, docked structures of D9 scFv are in green, and the acidic region of FV is in red. In panel (**C**), FX is in grey, acidic region of FV is in red, and prothrombin is in blue.

In silico approaches have been frequently proposed as methods to obtain information on the binding of mAbs toward their final targets [24,25]. Thus, we have used this approach to study the interaction of D9 scFv with FV. The structure of D9 scFv is made up of a canonical β-sandwich immunoglobulin topology. The FV structure has been solved at 3.3 Å resolution by Di Cera’s group using cryo-electron microscopy [26]. The structure revealed the entire A1-A2-B-A3-C1-C2 architecture with a disordered B domain (Figure 4A). The A1 domain (residues 1–316) is connected to A2 (residues 317–709) by a short basic segment (residues 304–316), while A2 is linked to B2 (residues 710–1545) through the acidic segment constituted by residues 657–709. B2 is connected to A3 (residues 1546–1877), which, together with C1 (residues 1878–2036) and C2 (residues 2037–2196), is part of the light chain of FV that is formed upon removal of the B2 domain by proteolysis [27]. Eight of the first ten poses of the docking study are reported in Figure 4B. The potential structures of the D9 scFv/FV complex obtained by the docking study were compared to that of the prothrombin-prothrombinase complex, reported in Figure 4C [28]. Docking analysis reveals that the A2 domain is the portion of the FV structure that has to be considered as a potential target for D9 scFv. In particular, D9 could link part of the acidic segment, which contains the residues involved in the prothrombin binding and prothrombinase function [21,29,30], but further studies are needed to verify this result. In this respect, it should be noted that computational data reveal a possible competition between D9 and prothrombin.

### 2.4. Generation and Characterization of the Novel Anti-FVa D9 Full-Size mAb

**Figure 5:**
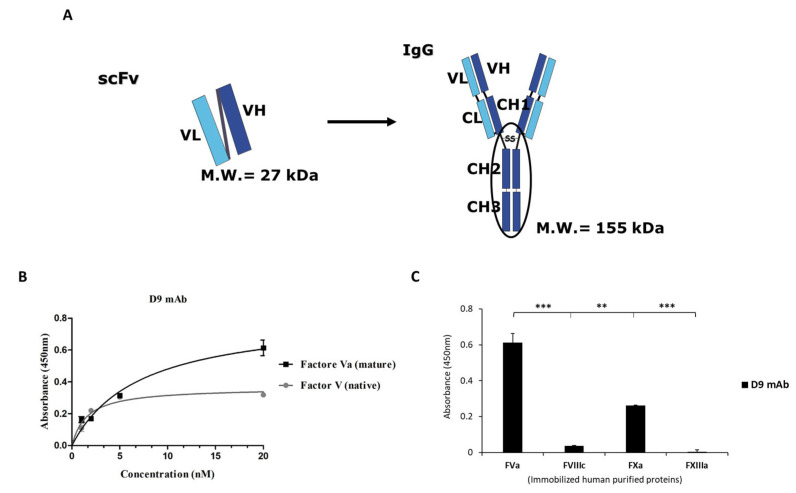
Binding of D9 mAb to FV and its cross-reactivity with other coagulation factors. (**A**) Schematical representation of the conversion of scFv into a fully human monoclonal antibody. (**B**) Binding curves of D9 mAb (1–20 nM) to immobilized coagulation Factor V native precursor (grey circles) and matured Factor Va (black squares) by ELISA assays. Regression analyses for generating the best-fit curve of the binding data were performed by using GraphPad Prism software (version 10.0.0) and reported in Supplementary Materials as Figure S1. K_D_, Bmax values, and normal distribution of the residuals are reported in the Supplementary Materials as Table S3. (**C**) Evaluation of the cross-reactivity of D9 mAb by ELISA assays for different coagulation factors coated on the plate at a fixed concentration of 5 µg/mL. D9 was detected by using an anti-Fab HRP-conjugated antibody (from Abcam, Cambridge, UK). Binding values were reported as the mean of two or three determinations. Data are presented as mean ± SD. The Shapiro–Wilk test was used to test the normality assumption by GraphPad Prism software (version 10.0.0), and *p*-values were calculated by comparing binding values of D9 mAb to immobilized FVa protein to that observed on FVIIIc, FXa, or FXIIIa protein, respectively. The values reported are *** *p* ≤ 0.001 and ** *p* < 0.01, which were obtained by Student’s *t*-test (two variables). Supplementary information (raw data) for Figure 5C is reported in the Supplementary Materials as Table S4.

To obtain a bivalent full-size mAb, the isolated scFv was converted into a more stable antibody format (IgG1) by subcloning the variable domains in the frame to human IgG1 Fc into mammalian expression vectors (Figure 5A). The recombinant antibody was produced and purified from the conditioned medium of CHO transfected cells by protein G affinity chromatography, as described in the Materials and Methods.

Once purified, D9 mAb was first tested by using ELISA assays at increasing concentrations on both the mature FVa and its native precursor FV in order to confirm its specific binding to the target even after the conversion into full-size mAb. The results, as shown in Figure 5B, indicate that the D9 monoclonal antibody was able to efficiently recognize the mature FVa and its native precursor FV, with an apparent affinity (K_D_) of 3–4 nM. To test the cross-reactivity of D9 mAb for other coagulation factors, FVa, FVIIIc, FXa, and FXIIIa were immobilized on multiwell plates, and D9 was tested by parallel ELISA assays at the concentration of 20 nM, which was the saturating concentration for the binding to FVa. D9 showed a slight binding to the FXa, whereas no binding at all was observed to FVIIIc and FXIIIa (Figure 5C), thus confirming its specificity for FV.

**Figure 6:**
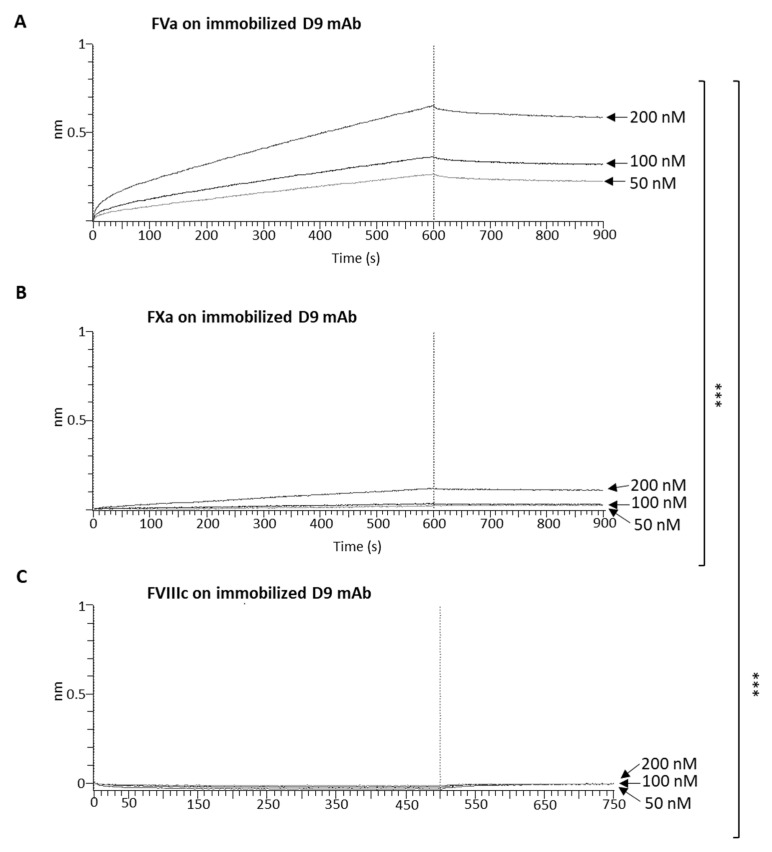
Binding kinetics of D9 by BLI analyses. (**A**) Binding of Factor Va tested as an analyte at increasing concentrations (50–200 nM) on the immobilized D9 mAb (used as a ligand) at a concentration of 2 µg/mL. (**B**) Binding of Factor Xa or (**C**) Factor VIIIc on the immobilized mAb performed in parallel assays under the same conditions. The sensorgrams show the association and dissociation rates of the analytes. The Shapiro–Wilk test was used to test the normality assumption; thus, *p*-values were calculated by comparing the nm shift generated by the interaction of FVa analyte with immobilized D9 to that observed for the interaction of FXa or FVIIIc analytes, respectively [31]. The value reported is *** *p* ≤ 0.001, which was obtained by Student’s *t*-test (two variables). Data used for statistical analysis are reported in the Supplementary Materials as Figure S2 and Table S5.

To further characterize the binding properties of the novel D9 monoclonal antibody, we performed Biolayer Interferometry (BLI) analyses to accurately evaluate the binding kinetics of D9 to the FVa and FXa coagulation factors. Despite the absence of binding observed in the ELISA assays, we also decided to test the binding of D9 to the FVIIIc again due to the significant sequence homology between FVa and FVIIIc in order to definitively exclude a potential cross-reactivity by performing a different type of assay. To this aim, D9 was immobilized on the protein A (ProA) biosensors at a concentration of 2 µg/mL; then, the binding of increasing concentrations of FVa (50–200 nM) was measured. The same experiment was performed in parallel by using FXa and FVIIIc in the same experimental conditions. As shown in Figure 6A, D9 showed a significant binding to the FVa, with a K_D_ of 1.162 × 10^−8^ M (≃12 nM), whereas a poor binding was detected to the FXa protein even when the coagulation factor was tested at the highest concentration of 200 nM (Figure 6B). No binding at all was detected for FVIIIc (Figure 6C). The difference in the K_D_ value observed by BLI compared to that measured by ELISA is due to the single-site (monovalent) binding affinity measured by BLI with respect to the bivalent one analyzed by ELISA, which is also affected by avidity.

### 2.5. Effects of D9 on the Complex Formation Between FVa and FXa

**Figure 7:**
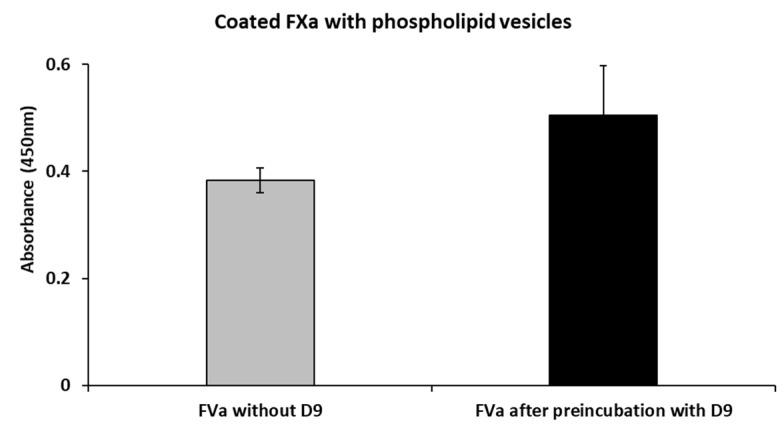
Competitive ELISA assay to test the effects of D9 in the formation of the FVa-FXa complex. The coagulation Factor Xa was coated on the plate at a concentration of ~2 µg/mL in the presence of phospholipid vesicles. Then, FVa at a concentration of 200 nM alone (grey bar) or after preincubation with D9 (5:1 mAb molar excess) (black bar) was added. Binding values were reported as the mean of at least two determinations. Data are presented as mean ± SD. Supplementary information is reported in the Supplementary Materials as Table S6.

In order to investigate the mechanism of action of this newly generated mAb, we performed competitive ELISA assays to test the effects of D9 on the binding of FVa to FXa in the presence of phospholipids added in order to increase the interaction between the two factors, as previously reported in the literature [32,33]. To this aim, FXa mixed with the phospholipids vesicles was coated on the plate; then, FVa, alone or preincubated with 5-fold molar excess of D9 for 1 h and 30 min at room temperature (RT), was added. The results, as shown in Figure 7 and Table S6, indicate that D9 does not significantly interfere with the binding between FVa and FXa.

### 2.6. Effects of the D9 mAb on the Coagulation Cascade

After testing the binding properties of the novel D9 mAb, we wanted to evaluate its ability to affect the coagulation cascade in a functional assay. To this aim, D9 mAb was preincubated with plasma samples at increasing concentrations for 1 h at RT, and then the prothrombin time (PT) and partial thromboplastin time (aPTT) were measured. The results, as shown in Table 1A, indicate that D9 significantly prolonged the aPTT, whereas no significant effects were observed on PT; thus, we further investigated its effects on the intrinsic pathway by measuring the levels of the other factors involved. The data, which were obtained from one of the experiments, as represented in Table 1B, show that D9 reduced the levels of FV in a dose-dependent fashion. The addition of D9 to the plasma was also able to even more significantly reduce the levels of other coagulation factors such as FVIII, FIX, and FXI, in particular at the higher doses of 1 and 2 μM. These results suggest that the novel D9 mAb is able to interfere with the intrinsic pathway of the coagulation cascade.

**Figure 8:**
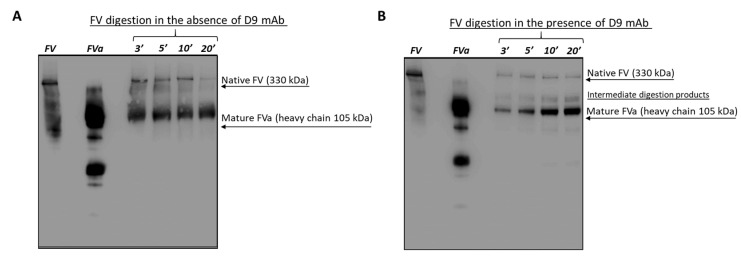
Time-course analysis by WB of Factor V digestion mediated by thrombin in the absence or in the presence of D9 mAb. The native FV protein was preincubated with D9 mAb (10-fold molar excess) in the presence of phospholipids, previously coated on a 96-well plate, for 90 min at RT; then, the proteolytic digestion with thrombin (1:500 thrombin:FV) was performed at increasing time intervals (3, 5, 10, 20 min) by incubating the mixtures at 37 °C. The mixture of digestion was stopped by adding the protease inhibitors and boiling the mixture for 5 min to inactivate the enzyme. The products of digested Factor V were measured by Western Blotting analysis by using an anti-FV monoclonal antibody to detect the signals. Untreated FV and FVa proteins were used as a reference of molecular weight. The panels (**A**) (in the absence of D9) and (**B**) (in the presence of D9) show the effects of thrombin on FV for the indicated time intervals. The blot images were acquired using the ChemiDoc Imaging System, and the acquisition and optimization were performed using Image Lab software. The full-length blot images are reported in theadditional file.

To further investigate the mechanism of action of D9, we performed enzymatic digestion of the native FV protein mediated by thrombin in the absence or in the presence of D9 to test whether the antibody could inhibit or slow down its activation by proteolysis. To this aim, we performed a time-course assay by analyzing the digestion products of FV protein obtained by the incubation with thrombin at increasing time intervals (3–20 min) using Western Blotting. Native FV, untreated or preincubated with D9 mAb (10-fold molar excess) for 1 h at RT, was incubated with thrombin in the presence of coated phospholipids. The reaction was blocked at the indicated time intervals by adding protease inhibitors, followed by inactivation of the enzyme at high temperatures. The mixture of digested products of Factor V was analyzed by WB by using an anti-FV monoclonal antibody recognizing an epitope common to both the native FV and the heavy chain of activated Factor Va. As shown in Figure 8, the inactive precursor Factor V, visible as a band of 330 kDa molecular weight, is proteolytically digested to mature FVa (heavy chain of 105 kDa) in the presence of thrombin after 3 min, leading to the total digestion (disappearance of the band of 330 kDa) after 20 min in the absence of D9 (Figure 8A). However, in the presence of D9 (Figure 8B), the digestion seems to be slower by comparing the levels of native, intermediate, and mature products with those observed in the absence of D9, especially after 3 or 5 min, therefore suggesting that the monoclonal antibody is able to partially impair the digestion mediated by the enzyme, thus slowing down its activation in line with the prolonged aPTT observed in the previous experiment relative to the blood coagulation.

## 3. Discussion

Thrombosis due to altered homeostasis represents a cardiovascular disease leading to severe complications such as heart attacks, strokes, and peripheral vascular disease [1,2,3,4,5,6,7]. For these reasons, it represents one of the most common causes of mortality worldwide.

The primary objective of anticoagulant therapy is to prevent thrombosis without causing bleeding. Conventional treatment of thrombosis has been represented by non-specific agents, including vitamin K antagonists and heparins [9,10], gradually replaced by DOAC (direct oral anticoagulants), which are easier to use due to their better pharmacological profile and lack of need for surveillance [11,12,13]. Even though they have a better safety profile than non-specific agents, bleeding rates have been reduced but not completely suppressed [14]. The development of monoclonal antibodies targeting specific coagulation factors presents a promising approach to obtaining targeted and safer anticoagulant therapies.

Among the coagulation components, Factor V represents an optimal target since it is an essential cofactor in the prothrombinase complex, which plays a pivotal role in the conversion of prothrombin to thrombin, a crucial enzyme in the coagulation cascade [20,21].

In this study, we isolated and characterized a novel human single-chain variable fragment (scFv), named D9, which binds with high specificity to both Factor V and its activated form, Factor Va. By using phage display technology, we designed a selection strategy based on two parallel pannings on both native FV and activated FVa in order to obtain an scFv endowed with a good affinity for both forms.

Then, the isolated scFv was converted into a more stable, fully human monoclonal antibody (IgG1) by subcloning the variable domains in the frame to human IgG1 constant regions into mammalian expression vectors used to transfect CHO cells. The secreted antibody was purified by using a protein G affinity chromatography and fully characterized.

Indeed, D9 was tested for its binding properties by docking analysis, ELISA, and BLI, showing its ability to recognize FV in either its native or mature (FVa) form with a K_D_ of 3–4 nM for bivalent equilibrium binding of mAbs to the immobilized target. Further characterization of binding kinetics with BLI revealed that D9 mAb also shows a good affinity for monovalent single-site binding (K_D_ of 12 nM).

We also evaluated its cross-reactivity with other coagulation factors (FVIIIc, FXa, and FXIIIa) and found that the novel anti-FV mAb showed poor binding to the FXa and no binding at all to the FVIIIc and the FXIIIa, thus confirming its specificity for FV.

To investigate the mechanism of action of D9, we analyzed its ability to affect the binding of FV to FX or its activation by proteolytic maturation. Functional assays, carried out using competitive ELISA assays, indicated that D9 mAb does not interfere with the formation of the FVa-FXa complex, whereas it seems to slow down the activation of FV to FVa mediated by thrombin in enzymatic digestion assays, likely by protecting the regions bound from the proteolytic attack by thrombin. This result is also supported by a docking analysis showing that the D9 binding site is located within the A2 domain of FV and, in particular, in the acidic segment (residues 657–679) close to the residues involved in the prothrombin binding [28,30]. Thus, D9 can hinder the prothrombin/thrombin binding to FV and following digestion to FVa by competing for the same binding site or by binding to an overlapping sequence and generating steric hindrance for the interaction of FV and thrombin. In line with this result, we also found that D9 prolongs the activated partial thromboplastin time (aPTT) and reduces levels of intrinsic pathway coagulation factors (FVIII, FIX, and FXI), thus inducing a mild anticoagulant effect. The reason why D9 more efficiently affects the intrinsic pathway compared to the extrinsic pathway by slowing the generation of FVa and, consequently, the activation of thrombin is likely due to its multiple positive feedback loops occurring in the intrinsic pathway during the initiation and amplification phases [34,35]. Thrombin indeed activates three different upstream cofactors (FV and VIII, as well as FXI) that are all involved in the intrinsic pathway, whereas in the extrinsic pathway, the effect of thrombin on the upstream steps is limited [35].

These findings suggest that the D9 mAb could become a potential therapeutic tool for modulating coagulation processes by targeting FV/FVa in the treatment of coagulation disorders. One of the limitations of this study is the lack of comparison of D9 efficacy with other anticoagulants, which is also due to the absence of an FDA-approved antibody-based drug specific to FV or for therapy of thrombosis, as well as the lack of further analyses that should be carried out to validate its therapeutic potential. However, we plan to perform these studies in the future by including epitope mapping, tests of its effects on prothrombin activation, as well as assays on samples of FV Leiden patients [3], and in vivo studies to confirm its efficacy. Furthermore, it could become a precious tool for diagnostic applications when the detection of FV in biological samples is requested to highlight FV deficit in patients with hemorrhagic diseases [4] or for immunoaffinity chromatography purification of Factor V from blood samples to be used for therapeutic aims.

## 4. Materials and Methods

### 4.1. Antibodies, Human Recombinant Proteins, and Phospholipids

The following human recombinant proteins were used:

Native Human Coagulation Factor V (F5-5300H) and His-tagged Recombinant Human FVIIIc Protein (F8-048H) are from Creative Biomart, Shirley, NY, USA. Human Factor Va Native Protein (RP-43128), Human Factor Xa Native Protein (RP-43114), Human Factor XIIIa Native Protein (RP-43123), and Human alpha-Thrombin Native Protein (RP43100) were all purchased from Invitrogen, Rockford, IL, USA.

The human commercial antibodies used are reported below:

Monoclonal Antibody specific to Factor V (MA1-43005, Invitrogen, Rockford, IL, USA); HRP-conjugated anti-M13 Monoclonal antibody (27-9421-01, Cytiva, Wilmington, DE, USA); HRP-conjugated anti-cmyc antibody (130-092-113, Miltenyi Biotec, Bergisch Gladbach, Germany); HRP-conjugated anti-mouse IgG, (7076, Cell Signaling, Danvers, MA, USA); and anti-human IgG (Fab’)2 HRP-conjugated goat monoclonal antibody (ab87422, Abcam, Biomedical Campus, Cambridge, UK).

The phospholipids 1,2-Dioleoyl-sn-glycero-3-phosphocholine (DOPC, 4235-95-4) and 1,2-dioctadecenoyl-sn-glycero-3-Phosphoserine (DOPS, 90693-88-2) were all from Cayman Chemical, Ann Arbor, MI, USA).

### 4.2. Bacterial Strains, Culture Media, and Antibiotics

*E. coli* TG1 bacterial strain was used for the production of phages; *E. coli* SF110 bacterial strain was used for the expression of the scFvs as soluble proteins. Bacterial growth was carried out in the culture 2xYT-Medium media (A0981, PanReac by AppliChem, Darmstadt, Germany). The media were supplemented with 1% D-(+)-Glucose (G7528, Sigma-Aldrich T0440, St Louise, MO, USA). The antibiotics used were ampicillin (A7492) at a concentration of 100 μg/mL and kanamycin (A1493) at 25 μg/mL (all from AppliChem, Darmstadt, Germany).

### 4.3. Selection of scFv-Phage Clones

Phagemid particles were isolated (10^13^ cfu) from the TG1 transformed with the library by using the M13-K07 helper phage (18311-019, Invitrogen, Rockford, IL, USA), as previously described [22,23,36]. Phage particles were purified and concentrated by two steps of precipitation with ice-cold 20% PEG (043443.A3 Thermo Fisher Scientific, Waltham, MA, USA) containing 2.5 M NaCl and washed with 20 mL of sterile water. After an additional PEG/NaCl precipitation step, phages were resuspended in Phosphate-Buffered Saline (PBS), centrifuged at 12,000 rpm for 15 min at 4 °C, and stored at 4 °C until use.

For each round of selection, phages were first blocked with a solution of PBS/Milk 5% for 30 min at 37 °C. The first round of panning was carried out by incubating the phages for 2 h at 4 °C by gently rotation with the coated Human Factor V (20 µg/mL) on Nunc polypropylene tube. After extensive washes with PBS, the bound phages were eluted with 76 mM citric acid (pH 2.5) in PBS for 5 min and then neutralized with 1 M Tris-HCl (pH 8.0). The recovered phages were amplified by infecting *E. coli* TG1 cells. The phagemid particles obtained from amplification were used for two following rounds of selection, performed in parallel either on coated Human Factor V or Human Factor Va immobilized at a concentration of 20 µg/mL on polypropylene tubes. Phages were then collected, as described above, and stored at 4 °C until use [23,37].

### 4.4. Preparation of scFv-Phages for ELISA and Analysis of Positive Clones

A TG1 culture was infected with the eluted phages (after 3 rounds of selection) and plated on 2xYT/agar containing 1% glucose and ampicillin (100 μg/mL). Individual clones were picked up, transferred into 96-well plates, and grown in 100 μL of 2xYT medium containing 1% glucose and ampicillin (100 μg/mL) for 18 h by shaking at 37 °C. The scFv-phages were produced by superinfection with M13-K07 helper phage for 1 h at 37 °C, then centrifuged at 1200 rpm for 30 min at 4 °C to pellet the bacteria, and aliquots of 50 μL of scFv-phage containing supernatants were used for ELISA.

Positive clones identified by ELISA were inoculated in 10 mL of 2xYT containing 100 μg/mL of ampicillin and 1% glucose and cultured overnight by shaking at 37 °C before the isolation of plasmids by using Wizard^®^ Plus SV Minipreps DNA Purification System (A1330, Promega, Madison, WI, USA) [36]. To analyze the cDNAs encoding the single-chain variable fragments, phagemids were digested with NcoI (R0193) and NotI (R0189S) enzymes (all from New England Biolabs, Ipswich, MA, USA) and loaded on 1% agarose gel for electrophoresis. The nucleotide sequences encoding scFvs were determined by using suitable PCR primers in an internal facility (Ceinge, Napoli, Italy). The sequences were then analyzed by a software for DNA and amino acid editing and alignments (GENtle-software version 1.9.4, Magnus Manske, University of Cologne, Germany).

### 4.5. Soluble scFv Expression and Partial Purification

The vector containing the cDNA encoding the anti-FVa scFv was used to transform the competent bacterial strain of SF110 that was plated overnight at 37 °C on a 2xYT Agar Petri dish supplemented with 100 µg/mL ampicillin and 1% glucose. Then, the single colonies were picked up and inoculated at 37 °C in 2xYT medium containing ampicillin and glucose at the same concentrations indicated above until the OD600 reached 0.8. Cells were centrifuged at 3500 rpm for 15 min and resuspended in glucose-free 2xYT medium. The expression of soluble scFv was induced by adding isopropyl-1-thio-β-D-galactopyranoside (IPTG) (A4773, PanReac by AppliChem, Darmstadt, Germany) at a concentration of 1 mM in the *E. coli* SF110 culture, which was then grown at 25 °C overnight [22,36].

Cell cultures were centrifuged at 3500 rpm for 15 min, and a periplasmic extract was obtained by resuspending cells in a solution of BPER Bacterial Protein Extraction Reagent (78248, Thermo Fisher Scientific, Waltham, MA, USA) with 1X Protease Inhibitors (11873580001, Roche Diagnostics GmbH, Mannheim, Germany), according to the manufacturer’s recommendations.

### 4.6. ELISA Assays of D9 Soluble scFv

To confirm the binding ability of the novel anti-FVa D9 scFv, ELISA assays were performed on immobilized Human Coagulation Factor V or Human Factor Va. The NuncTM flat-bottom 96-well plate was coated with 5 μg/mL of Factor V or Factor Va Proteins (both from Invitrogen, Rockford, IL, USA) in a solution of 0.05 M NaHCO_3_ for 48 h at 4 °C. After blocking the coated 96-well plates with a solution of PBS/Milk 5% for 1 h at 37 °C, the D9 soluble scFv was diluted in PBS/BSA 3% and incubated for 2 h at RT by gently shaking. As a positive control, a commercial anti-Factor V mAb (MA1-43005 Invitrogen, Rockford, IL, USA) was used at a concentration of 200 nM. After the first incubation, extensive washes were carried out with PBS; then, the plate was incubated for 1 h at RT with HRP-conjugated anti-cmyc or anti-mouse HRP-conjugated antibody for the detection of soluble D9 scFv or commercial anti-FV mAb, respectively. After washes, the plates were incubated with 3,3′,5,5′-tetramethylbenzidine (TMB Sigma-Aldrich T0440, St Louise, MO, USA). Absorbance at 450 nm was measured by the Envision plate reader (Perkin Elmer, Waltam, MA, USA, 2102).

### 4.7. Conversion of scFv into Full-Size mAb

A synthetic gene containing the sequence encoding the variable region of the light chain of the scFv and the constant region of the human lambda chain was inserted in NotI and XhoI sites of the pCDNA 3.4 plasmid (A14697, Invitrogen, Rockford, IL, USA) to generate the pCDN3.4 Light Chain expression vector. Similarly, a synthetic gene containing the sequence encoding the variable region of the heavy chain of the scFv and the constant regions of the human IgG1 heavy chain was cloned in the NotI and XhoI sites of the pCDNA3.4 plasmid to generate the pCDNA 3.4 heavy-chain expression vector.

CHO cells were maintained in Dulbecco modified Eagle medium (21969035, DMEM, Gibco, Life Technologies, Paisley, UK) supplemented with 10% fetal bovine serum (F2442, Sigma-Aldrich, St. Louis, MO, USA), antibiotics (100 µg/mL penicillin and 100 µg/mL streptomycin, 15140122 Gibco), and 1 mM L-glutamine (25030081, Gibco). Cells were transiently transfected by using calcium phosphate. Briefly, 2.5 µg of each plasmid was added to 500 µL of solution A (250 mM calcium chloride). Then, 500 µL of solution B (1.4 mM sodium salt of H2PO_4−_, 140 mM sodium chloride, and 50 mM Hepes pH 7.05) was added, and the solution was briefly mixed by using a vortex. After 1 min, one mL of the solution was added to the cells, and the flask was gently shaken to ensure distribution of the transfection cocktail. After an overnight incubation, cells were collected, resuspended in serum-free medium (Opti-MEM, 31985070, Gibco), and cultivated for additional 48 h. Conditioned medium was then collected, and the secreted antibody was purified by using a protein-G Sepharose FPLC column.

### 4.8. Binding Specificity of D9 mAb by ELISA Assays

To check the ability of D9 mAb to recognize FV either in its native or mature (FVa) form, the converted mAb was used to obtain binding curves at increasing concentrations. To this aim, Human Factor V or Factor Va proteins were immobilized on 96-well plate at 5 µg/mL for 48 h at 4 °C, and then D9 mAb was incubated at increasing concentrations (1–20 nM) for 1 h at RT. After adding the HRP-conjugated anti-Fab antibody, the signal was detected by measuring the absorbance at 450 nm, and the binding curves were obtained by using Prism (GraphPad Prism 5, version 10.0.0) tool.

To verify the cross-reactivity of D9 mAb for other coagulation factors, parallel ELISA assays were performed on Human Factor Va, Human Factor Xa, Human Factor XIIIa or Human Factor VIIIc immobilized on 96-well plates at 5 µg/mL for 48 h at 4 °C. After blocking with PBS/Milk 5% for 1 h at 37 °C, the novel D9 mAb was incubated at a concentration of 20 nM for 1 h at RT. After extensive washes with PBS, the plate was incubated for 1 h at RT with HRP-conjugated anti-Fab antibody. Absorbance at 450 nm was measured by the Envision plate reader (Perkin Elmer, 2102, Waltam, MA, USA).

### 4.9. Phospholipid Vesicles Preparation

As previously reported [32,33,38], a mix of 60% DOPC and 40% DOPS phospholipids was prepared in Tris-HCl pH 7.4 (50 mM), NaCl (100 mM) buffer to generate liposome particles, followed by sonication performed by using Bioruptor^®^ Plus sonication device (B01020001, Diagenode, Denville, NJ, USA) for 6 cycles until the formation of phospholipid-mixing vesicles. The mix was diluted 1:10 in NaHCO_3_ buffer and coated on 96-well plates overnight at 4 °C before the binding or enzymatic digestion assays.

### 4.10. Competitive ELISA Assays

To test the ability of D9 mAb to interfere with the formation of the complex between FVa and FXa, competitive ELISA assays were performed in the presence of phospholipid vesicles, which were prepared as described above [32,38]. The vesicles were incubated with FXa at the concentration of 200 nM for 1 h at 37 °C, and then the mixture was diluted in NaHCO_3_ and used to coat a 96-well plate for 48 h at 4 °C.

The binding of FVa was tested before or after preincubation with the novel D9 mAb added at 5-fold molar excess for 1 h and 30 min at RT. The binding of FVa was detected by incubating the mixtures with mouse anti-human FV monoclonal antibody for 2 h at RT, followed by an anti-mouse IgG HRP-conjugated secondary antibody for 1 h at RT. Finally, TMB was added, and HCl 1N was used to stop the reaction. The absorbance values were measured at 450 nm.

### 4.11. Biolayer Interferometry (BLI) Analyses

To analyze the binding kinetics of the novel D9 mAb for Human Factor Va, Factor Xa, and Factor VIIIc, BLI analyses were performed by using the Octet R4 Protein Analysis System (Sartorius, Fremont, CA, USA). Biosensors carrying the protein A (18-5010, Octet ProA Biosensors, Sartorius, Fremont, CA, USA) were used to perform the assays by following the manufacturer’s recommendation, as previously reported [39].

Briefly, the ProA biosensors’ tips were hydrated for 15 min in 200 µL of Kinetic Buffer (KB) 10X (0.1% BSA, 0.02% Tween, in PBS 1X). Then, D9 mAb was loaded at a concentration of 2 µg/mL for a time interval of up to 200 s. After washing, the association step was carried out by dipping the biosensors for 600 s in a solution containing human FVa, FXa, or FVIIIc used as analytes at increasing concentrations (50, 100, 200 nM). Then, the dissociation step was performed in KB buffer 10X for 300 s, and finally, the biosensors were regenerated according to the manufacturer’s recommendations. The nm shift values were determined by BLI analyses as previously reported [31]. The obtained data were acquired and processed into the Octet Analysis Studio Software 13.0 [40,41].

### 4.12. Effects of D9 on Blood Coagulation

D9 mAb was preincubated with plasma samples (0.5 mL) at increasing concentrations (100 nM–2 µm) for 1 h at RT. Routine clotting time tests, such as activated partial thromboplastin time (aPTT) and prothrombin time (PT), and second-level hemostasis assays, encompassing Factors II (FII), FV, FVIII, IX (FIX), X (FX), XI (FXI), and XII (FXII), were then measured by the automated ACL Top 550 coagulometer (Instrumentation Laboratory Company, Bedford, MA, USA). Each factor activity was quantified by performing a modified PTT test: the plasma analyzed was diluted and added to a deficient plasma for the factor that would be studied. Correction of the clotting time of the deficient plasma is proportional to the correction (% activity) of that factor on the plasma analyzed, interpolated from a calibration curve. The calibration curves for the coagulation factors (reported in the new supplementary Figure S4) were generated by using plasmas with known factor concentrations, employing deficient plasma, calibrator plasma, PT-based (tissue thromboplastin) or aPTT-based reagents, calcium chloride, and buffers, with clotting times measured on an automated coagulometer ACL Top 550.

### 4.13. Effects of D9 on FV Activation

#### 4.13.1. Thrombin Mediated Digestion of FV in the Absence or Presence of D9

To investigate whether D9 mAb can inhibit or slow down the digestion of the native FV protein mediated by thrombin, time-course digestion assays were performed, and the products were analyzed by WB. To this aim, FV (7.5 pM), untreated or preincubated with D9 mAb, was used at a concentration of 75 pM (10-fold molar) in 50 mM Tris-HCl, pH 7.4, NaCl (100 mM), and CaCl_2_ (2.5 mM) buffer for 1 h at RT and incubated with thrombin used at the concentration of 0.015 pM (1:500 thrombin:FV). The reactions, carried out in 96-well plate in the presence of coated phospholipids (prepared as mentioned above), were stopped after increasing time intervals (3–20 min) at 37 °C [38]. The digestion was blocked by adding protease inhibitors and inactivating the enzyme by boiling the mixture for 5 min.

#### 4.13.2. Western Blotting Analysis (WB)

To analyze the digestion products released from FV activation by thrombin, aliquots of the incubation mix, in the absence or in the presence of D9 mAb, were analyzed by SDS-PAGE and WB analyses, which were carried out by incubating the filters with an anti-FV monoclonal primary antibody (recognizing an epitope in the heavy chain of activated Factor Va), followed by an HRP-conjugated anti-mouse secondary antibody. Immunoreactive bands were visualized by enhanced chemiluminescence, as previously reported [42], acquired at the ChemiDoc MP Imaging System (12003154, Bio-Rad Laboratories S.r.l., Segrate, MI, Italy). Optimization was carried out using Image Lab software.

### 4.14. Statistics

The values were reported as the mean of three or more independent measurements, and the data are presented as mean ± SD. Statistical analyses were carried out first by testing the normality assumption with the Shapiro–Wilk test and then by using Student’s *t*-test (two variables) or Kruskal–Wallis. *p* ≤ 0.001 ***, *p* < 0.01 **, and *p* < 0.05 * were considered statistically significant [43].

### 4.15. Docking Analysis

To perform the docking study, a 1:1 stoichiometry has been chosen. The structure of FV has been downloaded from the Protein Data Bank (accession code 7KVE [28]), the structure of D9 has been modeled by using SwissModel [44], and the structure of an anti-Mcl1 scFv (PDB code 6QB9, 72% sequence identity https://doi.org/10.1107/S2059798319014116) was used as a template. Docking was performed using pyDockWEB [45]. Eight out of the first ten predictions by the docking server have D9 scFv recognizing the A2 domain and, in particular, part of the acidic segment (residues 657–679). The potential structures of the FV/D9 complex have been compared with that of FVa/FXa complex bound to prothrombin [28]). Figures have been generated with PyMOL-1.8 (www.pymol.org).

## 5. Conclusions

We used phage display technology to isolate a new binder for the coagulation Factor V by performing parallel panning rounds on both FV and its active form, FVa. By ELISA screening of positive clones on both proteins, we identified a positive clone, named D9, with high binding affinity. The novel D9 scFv was converted into a full-size human IgG1 mAb, which confirmed its binding to both the precursor FV and the active FVa by ELISA assays and BLI analyses, whereas it showed low or no binding to the other coagulation factors. D9 did not interfere in the binding between FVa and FXa; however, it was able to lower the activation of FV mediated by thrombin in line with its binding region, predicted by docking, and its effects on aPTT time in plasma samples, which suggests an anticoagulant property. These data, altogether, indicate that D9 could be used either as a potential therapeutic agent for inhibiting coagulation or as a diagnostic tool for the detection of FV in biological samples.

## 6. Patents

The authors declare that an European patent relative to D9 mAb was recently filed.

## Figures and Tables

**Table 1:** Effects of D9 on blood coagulation. Functional assays on plasma samples to measure the effects of the D9 mAb on the coagulation cascade. Plasma samples of 0.5 mL were preincubated for 1 h at RT with increasing concentrations of D9 (100 nM, 1 µM, and 2 µM). (**A**) The effects of D9 on prothrombin time and the partial thromboplastin time (**B**) or different coagulation factors were measured in both percentages and seconds. An untreated plasma sample was used as a negative control. Standard deviations were ±5–10%. Calibration curves and additional data relative to Table 1 are reported in the Supplementary Materials as Figure S3 and Table S7. Statistical analyses were carried out using both Student’s *t*-test and Kruskal–Wallis by comparing the samples treated at 1 and 2 μM concentrations of D9 with the untreated one. *p*-values are *p* < 0.01 ** and *p* < 0.05 *, which were considered statistically significant. ns means not significant.

**(A) D9 mAb effects on the coagulation time**														
**Sample**			**PT** **(v.r. 0.8–1.20 Ratio)**		**PT seconds**		**PT INR** **(v.r. 0.8–1.20 Ratio)**		**aPTT** **(v.r. 0.8–1.20 Ratio)**			**aPTT seconds**		
NORMAL POOL PLASMA (untreated)			1.01		11.5		1.01		1.02			29.3		
NORMAL POOL PLASMA (+100 nM D9 mAb)			0.98		11.2		0.97		1.07			30.6		
NORMAL POOL PLASMA (+1 μM D9 mAb)			0.95		10.9		0.95		**1.47**			**42.4**		
NORMAL POOL PLASMA (+2 μM D9 mAb)			0.96		11		0.96		**1.9**			**54**		
**(B) D9 mAb effects on the coagulation factors**														
**(% activity)**							**(seconds)**							
NORMAL POOL PLASMA	**FII** (v.r. 60–120%)	**FV** (v.r. 60–120%)	**FVIII** (v.r. 50–130%)	**FIX** (v.r. 50–120%)	**FX** (v.r. 60–120%)	**FXI** (v.r. 50–120%)	**FXII** (v.r. 50–120%)	**FII**	**FV**	**FVIII**	**FIX**	**FX**	**FXI**	**FXII**
(untreated)	95	102	84	109	96	98	83	14.2	16.9	53.5	51.3	21	55	50.2
(+100 nM D9 mAb)	105.6	97	73	97	96	94	77	13.9	17.1	54.6	52.2	21	55	51
(+1 μM D9 mAb)	101	82	53	67	96	57	61	14.1	17.8	58	55.2	21.3	59.6	53
(+2 μM D9 mAb)	98	73	40	47	93	40	49	14.2	18.3	60.3	58.3	21.6	64.3	54.5
*p*-value	ns	**	**	**	ns	**	**	ns	**	**	**	ns	**	*

## Data Availability

All data generated or analyzed during this study are included in this published article and its supplementary information files.

## Supplementary Materials

The following supporting information can be downloaded at https://www.mdpi.com/article/10.3390/ijms26062721/s1.

## Abbreviations

The following abbreviations are used in this manuscript:

aPTT	Activated partial thromboplastin time
BLI	Biolayer Interferometry
DOACs	Direct oral anticoagulants
DOPC	1,2-dioleoyl-sn-glycero-3-phosphocholine
DOPS	1,2-dioctadecenoyl-sn-glycero-3-phosphoserine
EMA	European Medicines Agency
FDA	Food and Drug Administration
FII	Factor II
FIX	Factor IX
FV	Factor V
FVa	Activated Factor V
FVIIIc	Factor VIIIc
FXa	Activated Factor X
FXI	Factor XI
FXII	Factor XII
FXIIIa	Activated Factor XIII
HRP	Horseradish peroxidase
IPTG	Isopropyl β-d-1-thiogalactopyranoside
KB	Kinetic Buffer
LMWH	Low-molecular-weight heparins
mAbs	Monoclonal antibodies
ProA	Protein A
PT	Prothrombin time
scFv	Single-chain variable fragment
VKA	Vitamin K antagonists
WB	Western Blotting
