# Supplementary material for: A Novel Human Anti-FV mAb as a Potential Tool for Diagnostic and Coagulation Inhibitory Approaches
Source: Int J Mol Sci. 2025 Mar 18;26(6):2721. doi: 10.3390/ijms26062721 (PMC11943385; doi:10.3390/ijms26062721)
Supplement: Supplementary file 1 [file ijms-26-02721-s001.zip › ijms-3462311-supplementary.pdf]

Title: A Novel Human Anti-FV mAb as a potential Tool for Diagnostic and Coagulation Inhibitory Approaches

Authors: Margherita Passariello[1,2], Rosa Rapuano Lembo[1,3], Lorenzo Manna[1,2], Ciro Miele[4], Antonello Merlino [5], Cristina Mazzaccara[4], Antonio Leonardi[2], Claudia De Lorenzo [1,2,*]

[1] Ceinge—Biotecnologie Avanzate s.c.a.r.l., via Gaetano Salvatore 486, 80145 Naples, Italy; margherita.passariello@unina.it (M.P.); rosa.rapuano@unimi.it (R.R.L.); lorenzo.manna@unina.it (L.M.)

[2] Department of Molecular Medicine and Medical Biotechnologies, University of Naples "Federico II", Via S. Pansini 5, 80131 Naples, Italy

[3] European School of Molecular Medicine, University of Milan, 20122 Milan, Italy

[4] UOC Laboratory Medicine of Hematology and Hemostasis, Federico II University Hospital, 80131 Naples, Italy; ciro.miele@unina.it (Ciro Miele)

[5] Department of Biology, University of Naples "Federico II", Complesso Universitario Monte Sant'Angelo, Via Cinthia, 26 Napoli, Italy; antonello.merlino@unina.it

[*] Correspondence: : cladelor@unina.it; Tel.: +39-081-3737868

Supplementary Materials and Figures

Supplementary information (raw data) for Figure 2 of the main Manuscript

Table S1

Assay I			*Assay II*		
Immobilized protein	*D9 phage clone (absorbance values)*	*anti-M13-HRP (background)*	Immobilized protein	*D9 phage clone (absorbance values)*	*anti-M13-HRP (background)*
**FV**	0.85<br>1<br>1.55		**FV**	0.77<br>1<br>1.3	0.019<br>0.015<br>0.015
**FVa**	2<br>2.3<br>1.9	0.019<br>0.015<br>0.02	**FVa**	1.7<br>1.1<br>1.3	0.029<br>0.015<br>0.017
**Fc**	0.049<br>0.039<br>0.038		**Fc**	0.029<br>0.018<br>0.039	0.02<br>0.01<br>0.015

Table S1. The table reports the absorbance values obtained by three independent measurements from two different experiments (n=6), referring to the ELISA assay obtained by testing the binding of D9-positive phage-scFv clones to the indicated immobilized proteins (Figure 2 of the main manuscript). The values corresponding to the background obtained by the HRP-conjugated anti-M13-antibody used for detecting the signals were also reported in the table. As a negative control, Fc protein was used.

Supplementary information (raw data) for Figure 3B of the main Manuscript

Table S2

<table>
<tr><th>Immobilized protein</th><th>D9 soluble scFv (not induced)</th><th>D9 soluble scFv (IPTG-induced)</th><th>anti-M13-HRP (background)</th><th>anti-FV mAb (positive control on FV)</th></tr>
<tr><td>FV
(preparation 1)</td><td>0.504
0.526
0.53</td><td>1.1
1.15
1.25</td><td>0.034
0.044
0.044</td><td rowspan="2">2.3
2.4
2.2</td></tr>
<tr><td>FVa
(preparation 2)</td><td>0.152
0.17
0.161</td><td>0.373
0.35
0.38</td><td>0.013
0.021
0.017</td></tr>
</table>

Table S2. The table reports the absorbance values obtained by three independent measurements that refer to the ELISA assays performed to test the periplasmic extracts (preparations 1 and 2) on immobilized human FV or FVa-purified protein, respectively (see Figure 3B of the main manuscript). The binding was detected by using the HRP-conjugated anti-M13 antibody, corresponding to the background reported in the table. As a positive control, a commercial anti-FV mAb was used.

Supplementary information (regression analysis of the binding data) for Figure 5 of the main Manuscript

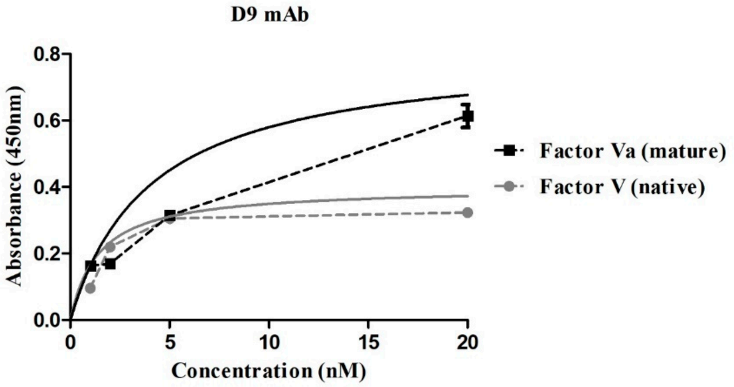

Table S3

Nonlin fit of binding data (regression analysis)	*Factor V (native)*	*Factor Va (mature)*
**One Site—Specific Binding**		
**Best-fit values**		
BMAX	0.3989	0.8133
KD	1.421	4.031
Std. Error		
BMAX	0.02547	0.07906
KD	0.3291	1.067
**95% Confidence Intervals**		
BMAX	0.3366 to 0.4613	0.6198 to 1.007
KD	0.6153 to 2.226	1.421 to 6.642
**Goodness of Fit**		
Degrees of Freedom	6	6
R^2	0.9075	0.9107
Absolute Sum of Squares	0.005800	0.02410
Sy.x	0.03109	0.06338
**Normality of Residuals**		
D'Agostino & Pearson omnibus K2	4.450	1.641
P value	0.1081	0.4402
**Number of points**		
Analyzed	8	8

Table S4

Immobilized protein	*D9 mAb (absorbance values)*
**FVa**	0.638<br>0.569<br>0.568
**FVIIIc**	0.036<br>0.037<br>0.039
**FXa**	0.2634<br>0.2614<br>0.2754
**FXIIIa**	0.01<br>0<br>0.011

Figure S1. Best-fit curves of binding data of D9 mAb to immobilized FV (dashed grey line) and FVa (dashed black line) were obtained from regression analyses performed by using GraphPad Prism software.
Table S3. The table reports all the values determined by nonlinear regression analyses.
Table S4. The table reports the absorbance values (after background subtraction) obtained by three independent measurements by ELISA assays (see Figure 5C of the main manuscript) performed to evaluate the cross-reactivity of D9 mAb to the different immobilized coagulation factors. The signal was detected by using an anti-Fab HRP-conjugated antibody.

Supplementary information (optical thickness values) for Figure 6 of the main Manuscript

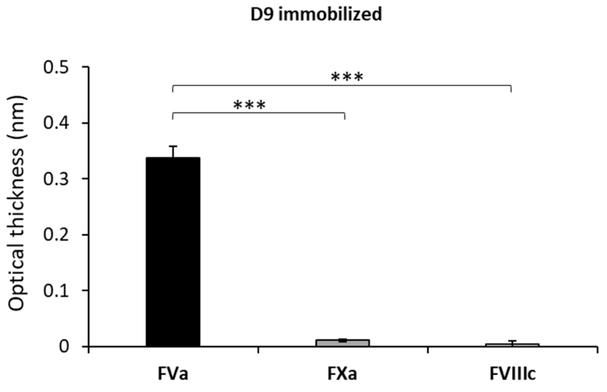

Table S5

Analytes tested on immobilized D9 mAb	(Optical thickness nm)	
**FVa**	0.3251<br>0.3262<br>0.3614	
**FXa**	*Assay I*	*Assay II*
	0.0120<br>0.0117<br>0.0081	0.0057<br>0.0092<br>0.0100
**FVIIIc**	0.0101<br>0.0019<br>0	

Figure S2. Optical data expressed as nm shifts were determined by BLI analysis by detecting the changes in the biomolecular interactions of the optical biolayer. The thickness values of FVa (black bar), FXa (grey bar), and FVIIIc (white bar) were reported in the bar graph as the mean of three determinations and presented as ± SD. The Shapiro–Wilk test was used to test the normality assumption; thus, p-values were calculated by comparing the optical values generated by the interaction of the FVa analyte with immobilized D9 to that observed for the interaction of FXa or FVIIIc analytes, respectively, at a concentration of 100 nM. The value reported is *** $P \leq 0{,}001$, which was obtained by Student's t-test (two variables).
Table S5. The table reports the optical thickness values, as nm shift, obtained by three or more independent measurements of the binding kinetics of D9 by BLI analyses (see Figure 6 of the main manuscript).

Supplementary information (raw data) for Figure 7 of the main Manuscript

Table S6

	Assay I		Assay II	
**Immobilized FXa with phospholipid vesicles**	*FVa detection (absorbance values)*	*anti-FV mAb used for detection (background)*	*FVa detection (absorbance values)*	*anti-FV mAb used for detection (background)*
**FVa without D9 mAb**	0.478<br>0.434<br>0.445	0.081<br>0.057	0.354<br>0.424	0.031<br>0.032
**FVa after preincubation with D9 mAb**	0.64<br>0.509<br>0.53		0.416<br>0.421	

Table S6. The table reports the absorbance values obtained by at least four independent measurements, referring to the ELISA assays performed by measuring the binding of FVa to the immobilized FXa before or after preincubation with D9 mAb (see Figure 7 of the main manuscript). The values corresponding to the background obtained by the anti-FV mAb used for the detection of FVa were also reported in the table.

Supplementary information for Table 1 of the main Manuscript (Calibration curves of all the factors involved in the intrinsic pathway)

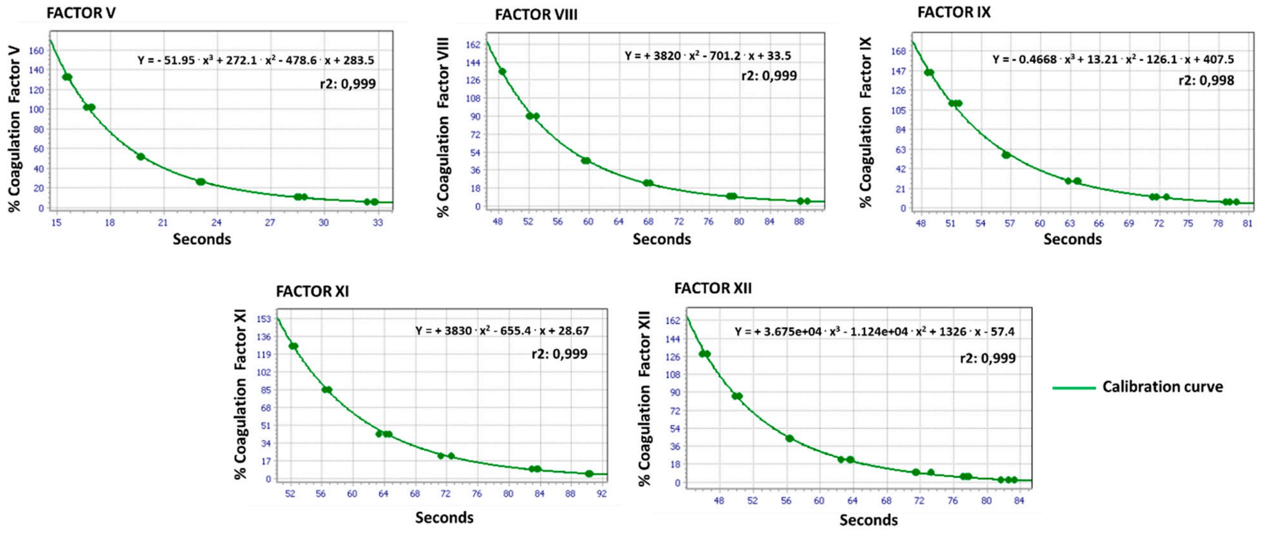

Table S7

Effect of D9 mAb on blood coagulation																						
**NORMAL PLASMA**	**PT Ratio**	**PT seconds**	**PT INR**	**aPTT seconds**	**aPTT Ratio**	**FII seconds**	**FII %**	**FV seconds**	**FV %**	**FVIII seconds**	**FVIII %**	**FIX seconds**	**FIX %**	**FX seconds**	**FX %**	**FXI seconds**	**FXI %**	**FXII seconds**	**FXII %**			
**Untreated**	1.01<br>1.02<br>1.00	11.5<br>11.6<br>11.4	1.01<br>1.02<br>1.00	29.4<br>29.1<br>29.3	1.01<br>1.02<br>1.03	14.2<br>14.3<br>14.2	96<br>94<br>94	16.9<br>16.3<br>16.5	102<br>113<br>107	53.5<br>53<br>54	82<br>86<br>84	51.5<br>51.1<br>51.5	112<br>107<br>109	20.8<br>21.1<br>21	98<br>94<br>95	55.3<br>54.7<br>55	96<br>101<br>97	50.1<br>50.3<br>50.2	84<br>83<br>83			
**(+100nM D9 mAb)**	0.97<br>1.00<br>0.97	11.1<br>11.4<br>11.1	0.97<br>1.00<br>0.97	31.3<br>30.2<br>30.2	1.08<br>1.06<br>1.06	13.9<br>13.7<br>13.9	104<br>110<br>103	17.1<br>16.4<br>16.6	97<br>110<br>105	54.5<br>54.6<br>54.9	74<br>73<br>73	52.4<br>52.2<br>52	96<br>98<br>97	21<br>21<br>21.2	96<br>96<br>95.8	56.5<br>54.9<br>55	86<br>100<br>96	51<br>50.8<br>51	77<br>78<br>77			
**(+ 1µM D9 mAb)**	0.96<br>0.94<br>0.95	11.1<br>10.7<br>10.8	0.96<br>0.94<br>0.95	43.4<br>42.2<br>41.8	1.46<br>1.48<br>1.47	14<br>14.2<br>14	101<br>102<br>96	17.8<br>17<br>17	82<br>95<br>95	57.4<br>58<br>58.2	54<br>53<br>52	55<br>55.7<br>55.1	67<br>65<br>70	18.1<br>21.2<br>21.3	97<br>93<br>92	59.6<br>59.1<br>60	57<br>68<br>62	52<br>53.3<br>53.3	61<br>60<br>60			
**(+2µM D9 mAb)**	0.95<br>0.96<br>0.97	10.8<br>11<br>11.1	0.95<br>0.96<br>0.97	53.7<br>53.6<br>53.6	1.95<br>1.88<br>1.88	14.1<br>14.2<br>14.1	98<br>96<br>99	18.3<br>17.9<br>17.7	73<br>77<br>75	60.3<br>60.6<br>62.4	41<br>42<br>35	58.5<br>58.2<br>58.3	45<br>49<br>48	21.6<br>21.3<br>21.6	89<br>93<br>93	62.8<br>65.9<br>64.3	42<br>37<br>42	56<br>54<br>53	49<br>50<br>45			

Figure S3. Calibration curves for the coagulation factor FV, FVIII, FIX, FXI, and FXII (see Table 1 of the main manuscript) were obtained as described: the calibration curves for the coagulation factors were generated by using plasmas with known factor concentrations, employing deficient plasma, calibrator plasma, PT-based (tissue thromboplastin), or aPTT-based reagents; calcium chloride; and buffers, with clotting times measured on an automated coagulometer ACL Top 550. R2 values were calculated, and the corresponding formulas were reported for each curve.

Table S7. The table reports the values, including measurements in seconds obtained by three independent measurements that refer to the assays on the effects of D9 on blood coagulation, reported in Table 1 of the main manuscript.
